# Supplementary material for: DYRK1A Up-Regulation Specifically Impairs a Presynaptic Form of Long-Term Potentiation
Source: Life (Basel). 2025 Jan 22;15(2):149. doi: 10.3390/life15020149 (PMC11856406; doi:10.3390/life15020149)
Supplement: Supplementary file 1 [file life-15-00149-s001.zip › life-3415068-supplementary.pdf]

Human genome (hg38)

Human YAC 152F7 (570 kb)

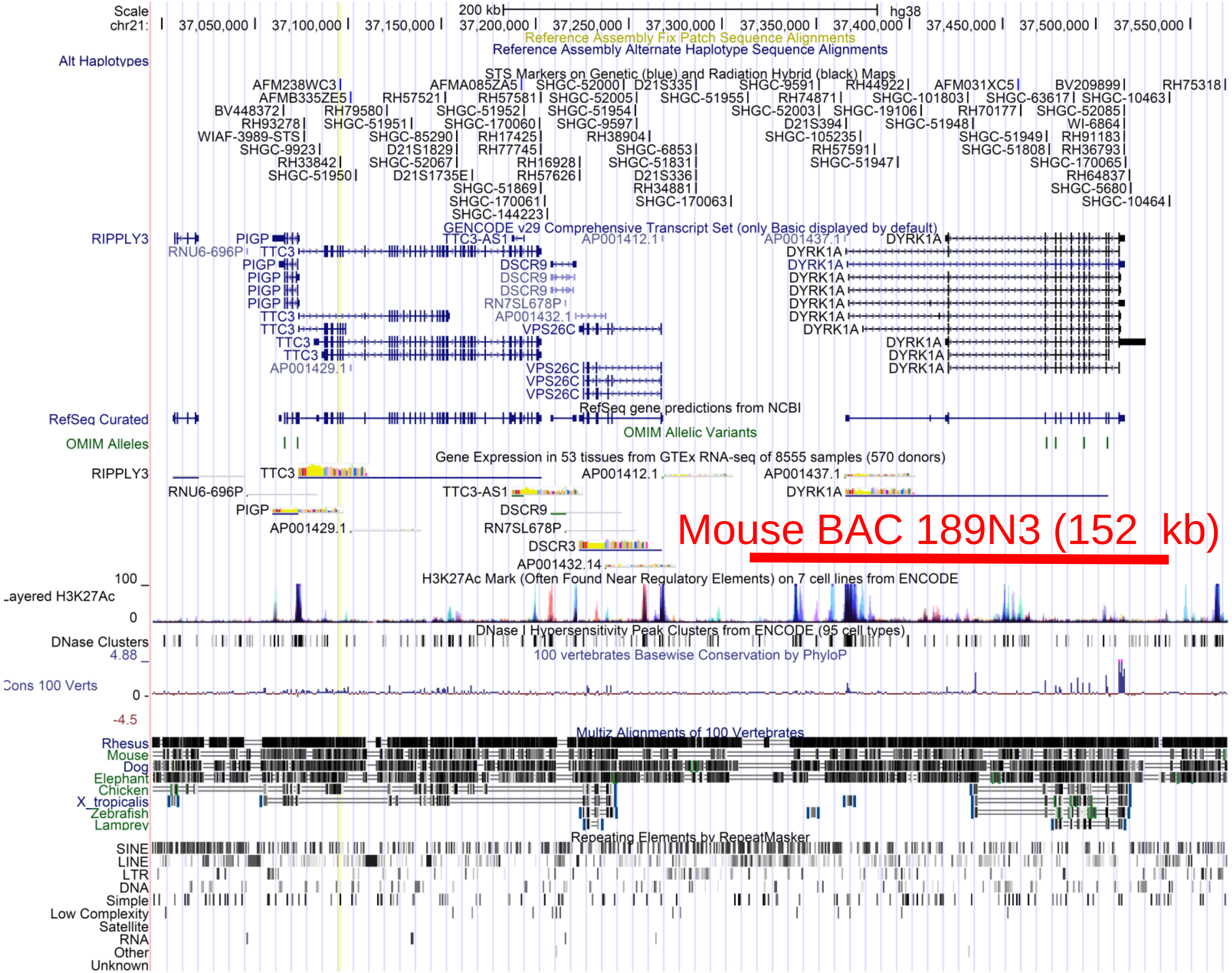

Suppl. Figure 1

Supplementary Figure 1. Schematic representation of the human YAC 152F7 transgene and of the mouse BAC 189N3 transgene.

We present here the genomic region of the human YAC 152F7 from the hg38 release (UCSC genome browser).

Note that human YAC 152F7 transgene (570 kb) involves a copy of six hChr21 genes: *RIPPLY3, PIGP, TTC3, DSCR9, VPS26C* and *DYRK1A* respectively.

In contrast, the mouse BAC 189N3 transgene consists of a 152 kb containing only the whole mouse Dyrk1a gene with a 6 kb flanking fragment on the 5' side and a 19 kb flanking fragment on the 3‘ (189N3 BAC clone from Research Genetics).

Mouse genome

Suppl. Figure 2

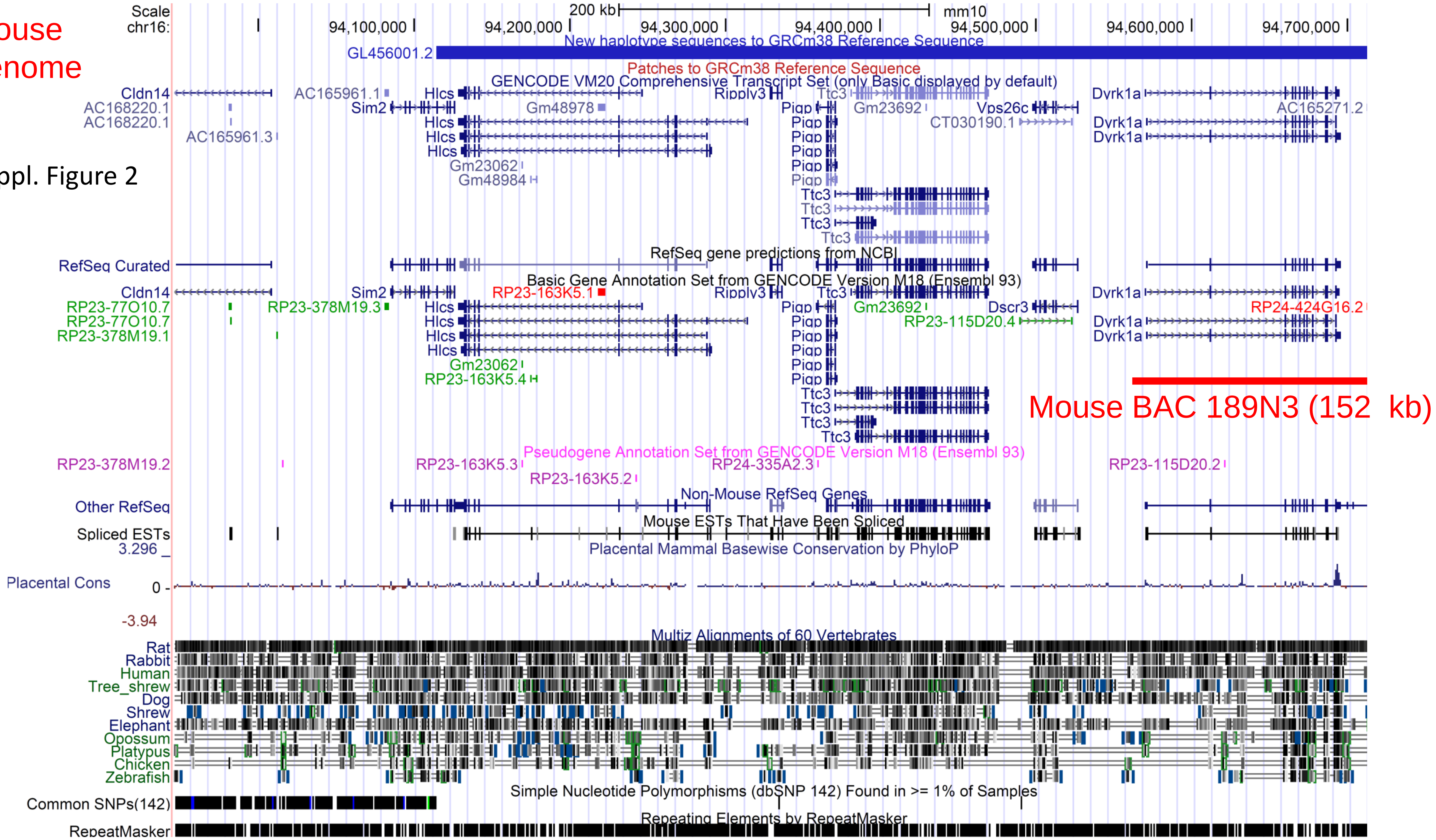

Supplementary Figure 2. Schematic representation of the mouse genomic region syntenic of the human YAC 152F7 transgene and that includes the mouse BAC 189N3 transgene.

We present here the mouse syntenic genomic region of the human YAC 152F7 (Mouse Dec. 2011 (GRCm38/mm10 release) (UCSC genome browser).

The YAC 152F7 transgene (570 kb) involves a copy of six hChr21 genes: *RIPPLY3, PIGP, TTC3, DSCR9, VPS26C* and *DYRK1A* respectively. Note that *DSCR9* is a primate-specific gene (22).

In contrast, the syntenic mouse region displays orthologous genes of *Ripply3, Pigp, Ttc3, DSCR9, Vps26c* and *Dyrk1a* in a more compact region of ~300 kb.

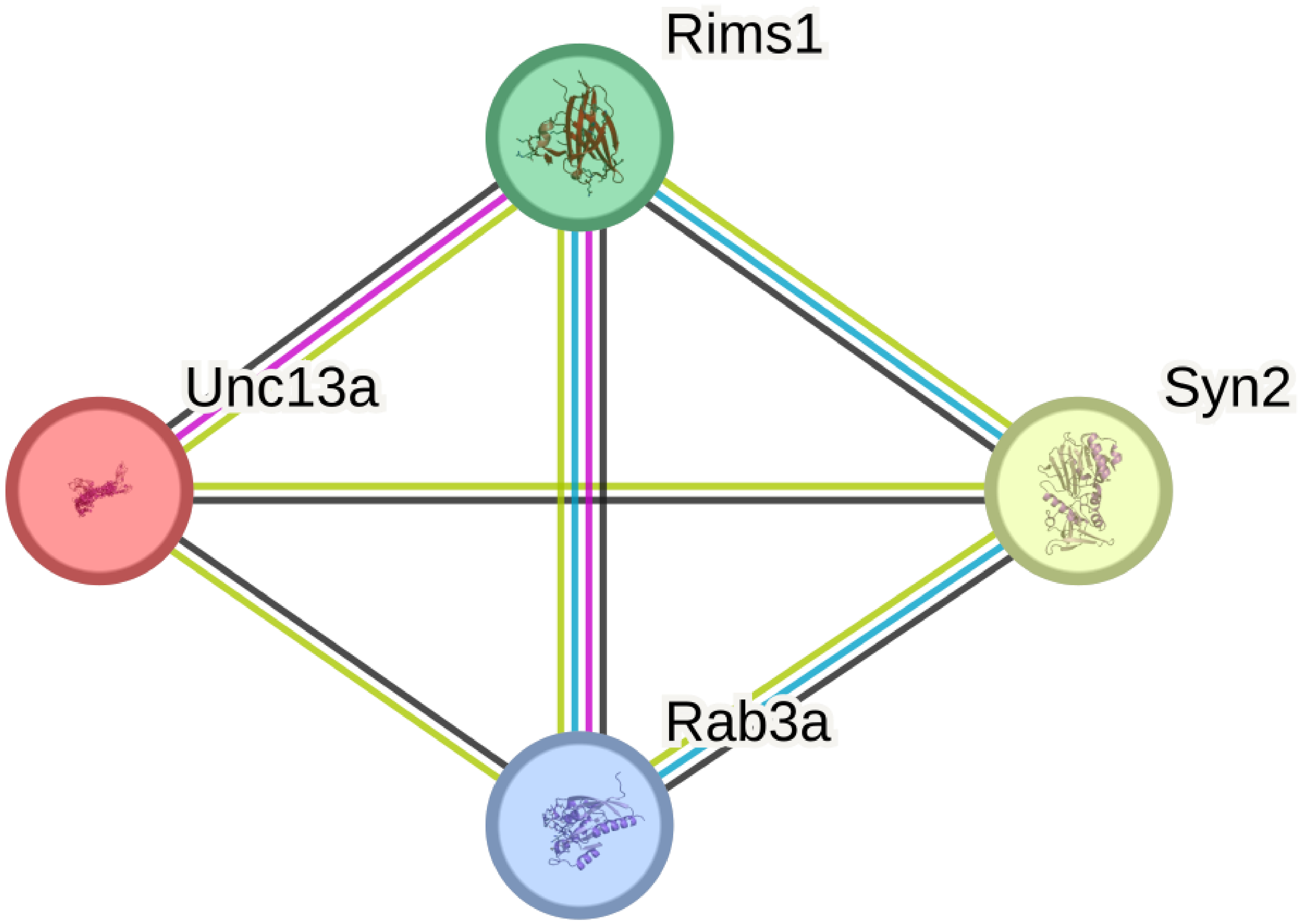

Suppl. Figure 3

Supplementary Figure 3. Interactions of RIMS1, SYN2, RAB3A and UNC13A.

RIMS1,SYN2, RAB3A and MUNC13A protein-protein interaction in mouse using String bioinformatics suite.
